# Restraint Stress Disrupted Intestinal Homeostasis via 5-HT/HTR7/Wnt/β-Catenin/NF-kB Signaling

**DOI:** 10.3390/ijms26094021

**Published:** 2025-04-24

**Authors:** Jiayu Yu, Zixu Wang, Yaoxing Chen, Yulan Dong

**Affiliations:** National Key Laboratory of Veterinary Public Health Security, College of Veterinary Medicine, China Agricultural University, Beijing 100193, China; yujy7288338@163.com (J.Y.); zxwang2007@163.com (Z.W.); yxchen@cau.edu.cn (Y.C.)

**Keywords:** 5-HT, Wnt/β-catenin, NF-kB

## Abstract

Stress may aggravate the development of inflammatory bowel disease and irritable bowel syndrome, in which the number of enterochromaffin (EC) cells and 5-hydroxytryptamine (5-HT) levels are abnormal, but the underlying mechanism remains largely unresolved. In this study, we discovered that restraint stress triggered the expression of Tph1, which led to 5-HT production. The 5-HT signaling then increased intestinal permeability, downregulated the expression of tight junction proteins, reduced the number of goblet cells and their ability to secrete mucin, promoted the expression of inflammatory cytokines, and ultimately damaged the intestinal mucosal barrier. Mechanistically, the 5-HT receptor HTR7 was highly expressed in the intestine. It interacted with 5-HT to initiate the Wnt/β-catenin signaling pathway, inducing an increase in intestinal EC cells and further promoting 5-HT secretion. Additionally, the activation of the Wnt/β-catenin signaling pathway could initiate the NF-κB signaling pathway and induce the expression of inflammatory cytokines. Blocking the 5-HT signal in mice inhibited the activation of the Wnt/β-catenin signal, thereby alleviating intestinal inflammation. Our findings revealed a novel role for 5-HT in intestinal inflammatory diseases and represent a potential new therapeutic target.

## 1. Introduction

Psychological stress is a frequent acute emotional reaction to perceived threats or challenges in one’s environment. It is often associated with disruptions to normal intestinal physiology. It is well known that psychological stress is the trigger of inflammatory bowel disease (IBD) [1,2] and irritable bowel syndrome (IBS) [3,4]. This conclusion has been demonstrated in human [5,6] and animal model [7,8] studies, where excessive stress increased inflammation [9], barrier disruption [10], reactivation of disease induced by chemical exposure [11], and increased susceptibility to enteric infection [12].

Intestinal epithelial cells (IECs) are composed of a variety of different cells, including goblet cells, Paneth cells, enteroendocrine cells, and stem cells, which work together to maintain barrier integrity and coordinate appropriate immune responses to both pathogens and the endogenous microbiota. Enterochromaffin (EC) cells are the best characterized subset of the enteroendocrine cells in the epithelial lining and the main site for the synthesis and release of 5-HT. 5-hydroxytryptamine (5-HT), also known as serotonin, plays a key role in many biological processes [13]. 5-HT exerts biological functions through engagement of 5-HT receptors. EC cells are dispersed in the epithelium of the mucosal layer of the gastrointestinal tract and release 5-HT from the top and bottom into the intestinal lumen in response to various mechanical and chemical stimuli [14,15]. Tryptophan hydroxylase (Tph) catalyzes the synthesis of 5-HT [16].

Hyperplasia of EC cells and dysregulation of 5-HT secretion by EC cells have been found in colons from IBS patients [17,18]. Abnormal alterations in EC cell numbers and intestinal 5-HT content have been observed in experimental colitis [19] and in the ulcerative colitis (UC) and irritable bowel syndrome (IBS) [20]. Research has found that the Tph1-deficient (Tph1^−/−^) mice, which have significantly reduced 5-HT levels in the gut, significantly ameliorates colitis severity in chemical induction models in chemically induced colitis [19]. Moreover, 5-HT plays a key role in activating immune cells to produce proinflammatory cytokines [19,21]. Stimulation of murine peritoneal macrophages with 5-HT induces the production of proinflammatory cytokines IL-1 and IL-6 in a nuclear factor κB (NF-κB)-dependent manner and enhances their phagocytic capacity [19,22]. Recent studies have found that 5-HT level is closely related to Wnt/β-catenin signaling activation in cancer models [23]. A large number of studies have found that there is crosstalk between the Wnt/β-catenin pathway and NF-κB pathway [24,25].

Despite the significant association between stress and gastrointestinal disorders, the interaction among 5-HT, Wnt/β-catenin and NF-kB is still unclear. To test this, we used a murine restraint stress model [26], and we found that restraint stress could induce the increase in intestinal 5-HT levels and damage the intestinal mucosal immune barrier. 5-HT could activate Wnt/β-catenin signaling through the HTR7 receptor, thereby promoting the differentiation of intestinal stem cells into intestinal EC cells and the additional secretion of 5-HT. In addition, Wnt/β-catenin signaling could regulate the activation of NF-kB pathway and promote the expression of inflammatory cytokines. Blocking 5-HT in mice could inhibit the activation of Wnt/β-catenin, thereby reducing intestinal inflammation. These findings provide insights into how 5-HT alters intestinal function and how this can help reduce the detrimental effects of stress on intestinal integrity. This also provided an important basis for the prevention and treatment of stress intestinal diseases in the future.

## 2. Results

### 2.1. Restraint Stress Induced the Increase in 5-HT Level, Leading to the Damage of Intestinal Barrier

**Figure 1:**
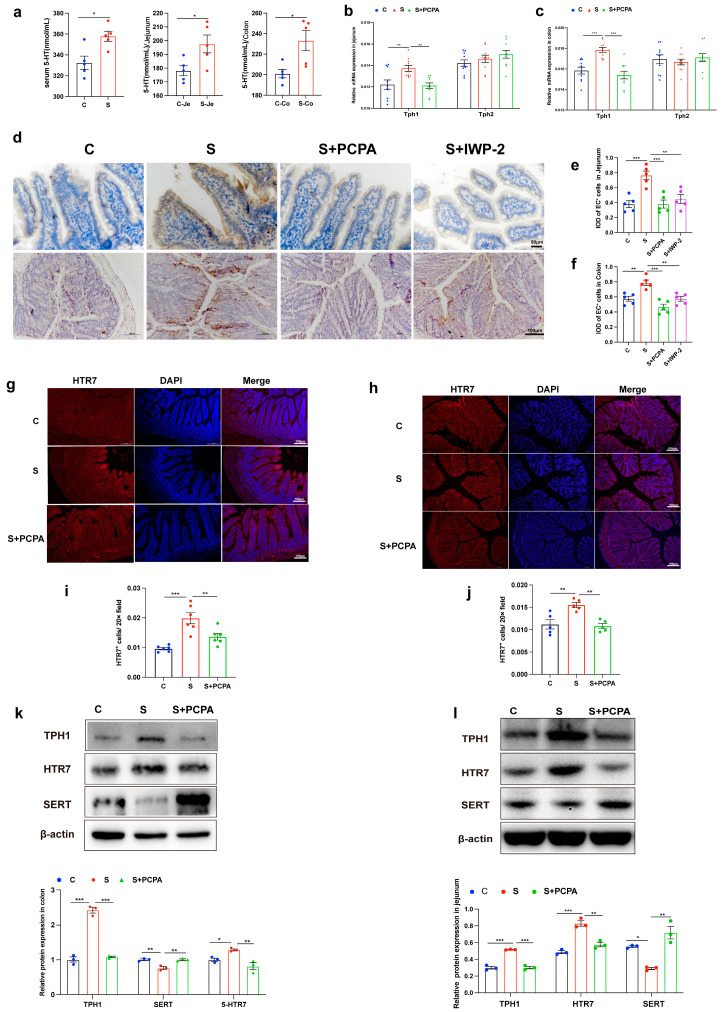
Restraint stress induced an increase in intestinal 5-HT levels. (**a**) Changes in 5-HT levels in serum and intestine of mice (n= 5). (**b**,**c**) Changes in Tph1 and Tph2 mRNA levels in jejunum and colon tissues of mice (n = 10). (**d**) EC^+^ cells in the jejunum and colon were detected by immunohistochemistry, and (**e**,**f**) the average integrated optical density (IOD) of the positive cells was measured by ImageJ 1.54p (n = 5). (**g**–**i**) HTR7 immunofluorescence staining and HTR7 cell intensities in jejunum and colon were analyzed by ImageJ. (**g**–**j**) The expressions of TPH1, HTR7, SERT and β-actin protein were examined in jejunum and colon by Western blot, and relative protein levels were normalized to β-actin (n = 3) (**k**,**l**). Each sample was assayed three times. Data are presented as the mean ± SEM. Differences were assessed by ANOVA and denoted as follows: * *p* < 0.05; ** *p* < 0.01; *** *p* < 0.001 indicate significant difference. C: control group; S: restraint stress; S + PCPA: restraint stress + PCPA; S + IWP-2: restraint stress + IWP-2.

Analysis following 14 consecutive days of restraint stress exposure on mice showed that the levels of 5-HT in serum and intestine were significantly increased, especially in the jejunum and colon (Figure 1a). To elucidate the cellular origins of this 5-HT surge, we focused on two principal endogenous sources in the gastrointestinal tract: EC cells, which express TPH1, and serotonergic neurons, which express TPH2. Both TPH1 and 2 are responsible for the biosynthesis of 5-HT [13]. Quantitative analysis revealed a significant upregulation of TPH1 expression at both transcriptional and translational levels in restraint-stressed mice (Figure 1b,c,k,l). In contrast, TPH2 expression remained unaltered (Figure 1b,c), indicating EC cells as the predominant contributors to stress-induced 5-HT production. Consistent with this hypothesis, immunohistochemical quantification demonstrated an increase in 5-HT-positive EC cell density in the jejunal epithelium of restraint-stressed mice (Figure 1d–f). To exclude potential confounding effects from altered 5-HT clearance, we analyzed the serotonin transporter (SERT). While SERT mRNA levels remained stable, protein expression decreased significantly (Figure S1e, Figure 1k–l), suggesting post-transcriptional regulation of SERT activity under stress conditions [27].

To delineate the downstream signaling pathways, we performed comprehensive profiling of 5-HT receptor subtypes.

**Figure 2:**
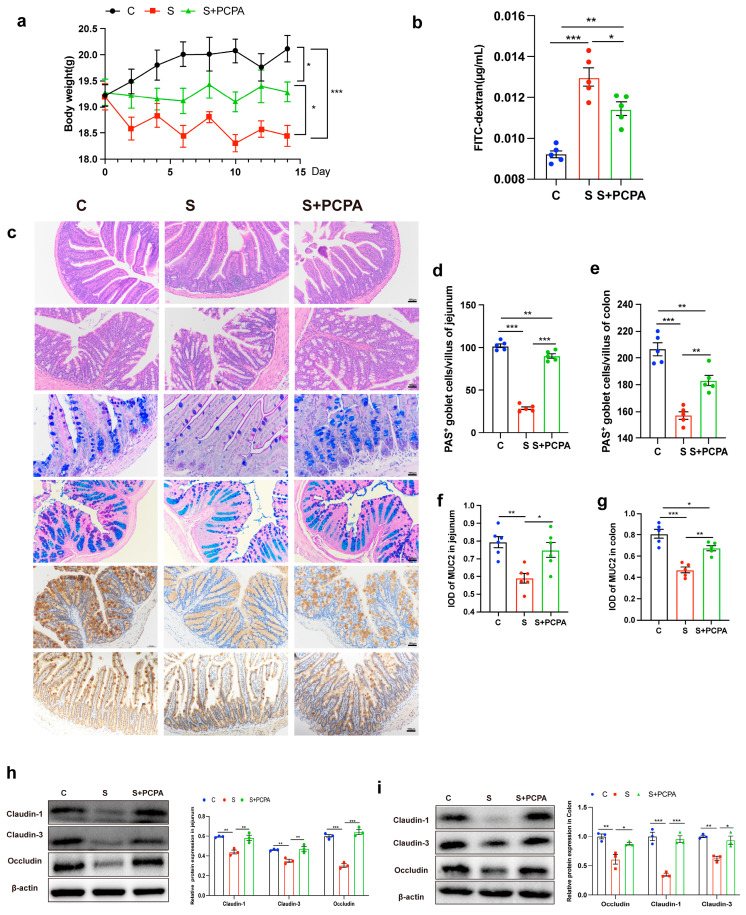
The damage of intestinal barrier induced by restraint stress was caused by 5-HT. (**a**) Daily body weight was monitored (n = 10). (**b**) Analysis of intestinal permeability using FITC-dextran in serum (n = 5). Histopathology (H&E), AB/PAS-stained mice in jejunum and colon showing the goblet cells (**c**) and the number of goblet cells was counted (**d**,**e**), immunohistochemical staining of MUC2 (**c**) in colonic tissue and the average integrated optical density (IOD) of the positive cells was measured by ImageJ (**f**,**g**). The scale bar represents 100 μm in H&E, AB/PAS and MUC2 staining. The expression of tight junction protein Occludin, Claudin-1, Claudin-3 and β-actin protein were examined in jejunum (**h**) and colon (**i**) by Western blot, and relative protein levels were normalized to β-actin (n = 3). Each sample was assayed three times. Data are presented as the mean ± SEM. Differences were assessed by ANOVA and denoted as follows: * *p* < 0.05; ** *p* < 0.01; *** *p* < 0.001 indicate significant difference. C: control group; S: restraint stress; S + PCPA: restraint stress + PCPA.

Strikingly, Htr7 mRNA was highly expressed in the jejunum and colon tissues of restraint-stressed mice, whereas Htr2, Htr3, and Htr4 showed no significant alterations in mRNA levels (Figure S1f,g). This transcriptional upregulation was corroborated at the protein level through dual approaches: (1) immunofluorescence imaging revealed intensified HTR_7_ signals predominantly localized to the epithelial layer (Figure 1g–j); (2) Western blot quantification confirmed a substantial increase in HTR7 protein expression (Figure 1k,l). Collectively, these findings establish a mechanistic cascade whereby restraint stress (1) enhances 5-HT biosynthesis through TPH1-mediated production in EC cells, (2) reduces SERT-mediated 5-HT clearance, and (3) selectively upregulates HTR7 receptor expression. This tripartite regulation suggests HTR7 as a key mediator of stress-induced serotonergic signaling in the gut, a hypothesis warranting further validation through in vitro receptor-specific functional studies.

To investigate the pathophysiological role of stress-elevated 5-HT in vivo, we employed a pharmacological intervention strategy using the TPH inhibitor parachlorophenylanaine (PCPA). Systemic TPH inhibition significantly reversed the restraint stress-induced serotonergic alterations: (1) downregulated TPH1 expression in jejunum and colon compared to stress group (Figure 1b,c,k–l); (2) reduced HTR7 expression to baseline levels at both mRNA (Figure 1h,i) and protein levels (Figure 1k,l) (3) restored SERT protein expression in colon (Figure 1k,l); (4) normalized EC cell density in jejunum and colon (Figure 1e–g).

PCPA treatment attenuated stress-induced body weight loss (Figure 2a). Using the FITC-dextran (4 kDa) intestinal permeability assay, we observed that PCPA reduced serum fluorescence intensity compared to restraint-stressed mice (Figure 2b). The results of HE staining showed that PCPA could alleviate the changes in intestinal histological structure caused by restraint stress (Figure 2c). It was also found by AB-PAS and MUC2 staining that restraint stress led to a decrease in the number of goblet cells and a disruption in the ability of goblet cells to secrete MUC2 in the jejunum and colon, but this phenomenon could be partially reversed after PCPA treatment (Figure 2c,d–g). The expression of tight junction protein Occludin, Claudin-1 and Claudin-3 in jejunum and colon tissues of mice decreased significantly after restraint stress, but returned to normal after adding PCPA (Figure 2h,i). In addition, by detecting the mRNA level changes of Muc2 (the main component of mucous layer), Tff3 (promoting mucosal repair and protection), and Klf3 (participating in the transcription of barrier function) in jejunum and colon tissues, it was found that the addition of PCPA alone did not affect the expression of these genes (Figures S2 and S3). Taken together, the disruption of intestinal barrier induced by restraint stress was mainly caused by 5-HT overexpression.

### 2.2. 5-HT Engagement Triggered Wnt/β-Catenin Signaling Activation

**Figure 3:**
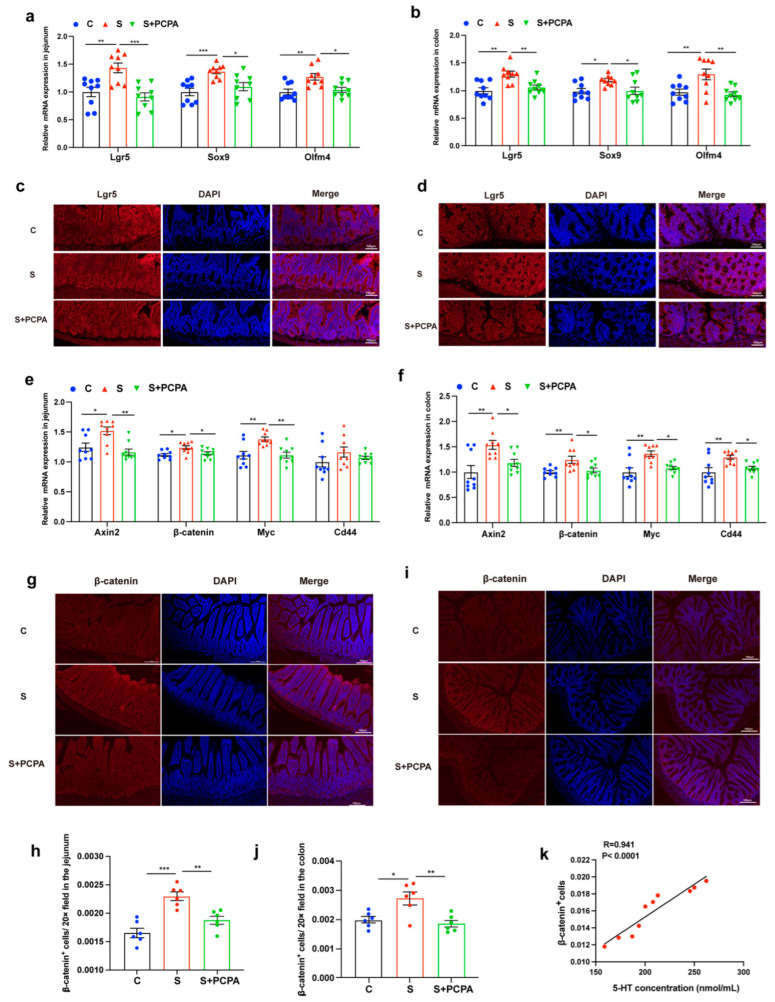
Restraint stress activated Wnt/β-catenin signaling through 5-HT. The expressions of Lgr5, Sox9, and Olfm4 were measured by real-time qPCR in jejunum (**a**) and colon (**b**) tissues of mice (n = 10). Lgr5 immunofluorescence staining in jejunum (**c**) and colon (**d**) tissues. The expressions of Axin2, β-catenin, Myc, and Cd44 were measured by real-time qPCR in jejunum (**e**) and colon (**f**) tissues of mice (n = 10). β-catenin immunofluorescence staining in jejunum (**g**) and colon (**i**) tissues and β-catenin^+^ cell intensities in jejunum (**h**) and colon (**j**) were analyzed by ImageJ. (**k**) Correlation between the expression of β-catenin-positive cells and 5-HT levels in intestinal tissue. Each sample was assayed three times. Data are presented as the mean ± SEM. Differences were assessed by ANOVA and denoted as follows: * *p* < 0.05; ** *p* < 0.01; *** *p* < 0.001 indicate significant difference. C: control group; S: restraint stress; S + PCPA: restraint stress + PCPA; S + IWP-2: restraint stress + IWP-2.

Our observation of increased enterochromaffin (EC) cell numbers under restraint stress prompted us to hypothesize that stress-induced intestinal stem cell (ISC) proliferation might drive excessive differentiation into EC cells. To investigate this mechanism, we first analyzed mRNA expression of key ISC markers (Lgr5, Sox9, and Olfm4) in jejunal and colonic tissues. Restraint stress significantly upregulated these stem cell markers compared to control conditions, but their expression levels returned to baseline following PCPA treatment (Figure 3a,b). Lgr5+ intestinal stem cells (ISCs) can differentiate into all cell types of the intestinal epithelium [28]. This finding was corroborated by Lgr5 immunofluorescence staining, which revealed increased proliferation of Lgr5+ ISCs in the area of intestinal crypts under stress conditions, with PCPA administration effectively suppressing this proliferative response (Figure 3c,d). Given that Lgr5+ ISCs serve as the primary source of intestinal epithelial renewal and function as Wnt agonist receptors [29], we subsequently examined Wnt/β-catenin signaling activation. Quantitative PCR analysis demonstrated that restraint stress significantly enhanced mRNA expression of canonical Wnt target genes (Axin2, Myc, and Cd44) in both jejunal and colonic tissues. Notably, PCPA treatment attenuated these stress-induced transcriptional changes (Figure 3e,f).

**Figure 4:**
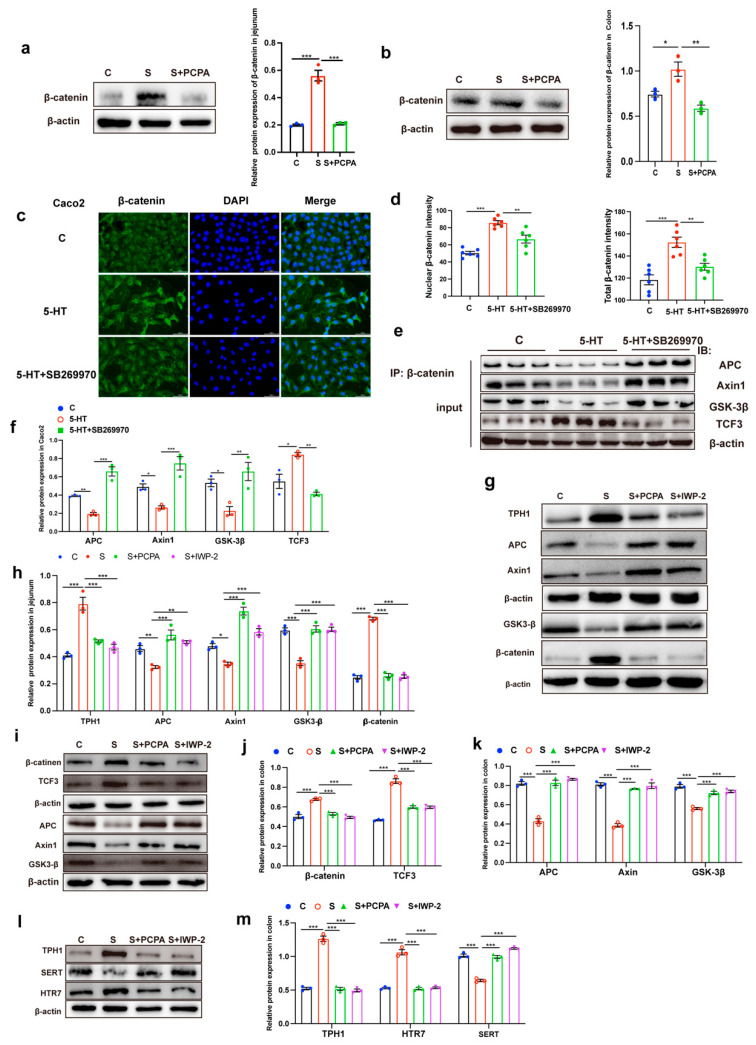
5-HT activated Wnt/β-catenin by acting on the HTR7 receptor. The expression of β-catenin and β-actin protein were examined in jejunum (**a**) and colon (**b**) by Western blot, and relative protein levels were normalized to β-actin (n = 3). Immunofluorescence staining of β-catenin in Caco2 cells (**c**) and total and nuclear β-catenin intensities were analyzed by ImageJ (**d**) (n = 6 hole). 5-HT and SB269970 treated Caco2 cells were used for β-catenin coimmunoprecipitation, input samples from the coimmunoprecipitation assay were analyzed by Western blot (**e**), and relative protein levels were normalized to β-actin (**f**) (n = 3). The expressions of TPH1, APC, Axin1, GSK3-β, β-catenin and β-actin protein were examined in jejunum (**g**) by Western blot, and relative protein levels were normalized to β-actin (**h**) (n = 3). The expression of β-catenin, TCF3, APC, Axin1, GSK3-β, TPH1, SERT, HTR7 and β-actin protein were examined in colon (**i**,**l**) by Western blot, and relative protein levels were normalized to β-actin (**j**,**k**,**m**) (n = 3). Each sample was assayed three times. Data are presented as the mean ± SEM. Differences were assessed by ANOVA and denoted as follows: * *p* < 0.05; ** *p* < 0.01; *** *p* < 0.001 indicate significant difference. C: control group; S: restraint stress; S + PCPA: restraint stress + PCPA; S + IWP-2: restraint stress + IWP-2.

β-catenin is the major effector protein of the Wnt/β-catenin pathway. At the protein level, we observed significant β-catenin accumulation in stressed intestinal tissues through both immunofluorescence staining (Figure 3g–j) and Western blot analysis (Figure 4a,b). Correlation analysis revealed a strong positive relationship between intestinal 5-HT levels and β-catenin-positive cell counts (Figure 3k), suggesting functional crosstalk between 5-HT signaling and Wnt pathway activation. Importantly, PCPA administration not only reduced 5-HT biosynthesis but also effectively normalized β-catenin protein expression in both intestinal segments (Figure 3g–j and Figure 4a,b). These findings collectively demonstrate that restraint stress activates the Wnt/β-catenin signaling cascade in a 5-HT-dependent manner, driving ISC proliferation and subsequent EC cell overproduction.

Building upon our in vivo findings that 5-HT signaling enhances β-catenin stability to activate the Wnt/β-catenin pathway, we next sought to mechanistically validate this relationship in vitro using the human intestinal epithelial cell line Caco2. Immunofluorescence analysis revealed that the total β-catenin level was increased after 5-HT treatment, especially in the nucleus (Figure 4c,d). This observation prompted investigation into 5-HT’s regulatory effects on β-catenin’s molecular interactions.

Given that β-catenin stability is principally controlled through phosphorylation by the APC/Axin/GSK-3β destruction complex, we first determined whether 5-HT influences β-catenin association with this degradation machinery. Co-immunoprecipitation experiments using a β-catenin-specific antibody demonstrated that 5-HT treatment significantly reduced interactions between β-catenin and core components of the destruction complex (Figure 4e,f). Conversely, 5-HT enhanced β-catenin functional engagement by promoting its interaction with the transcriptional coactivator TCF3 (Figure 4e,f).

We next investigated if antagonists of 5-HT receptors attenuated 5-HT-induced growth of β-catenin protein level. When 5-HT was added after SB-269970 (HTR7 antagonist) pretreatment for 2 h, we found enhanced interaction between β-catenin and the degradation complex (Figure 4e,f). These results indicated that 5-HT engagement triggered HTR7, thus activating Wnt/β-catenin signaling via blocking β-catenin degradation. IWP-2 is a specific inhibitor against Wnt/β-catenin activation that blocks Wnt signaling by inhibiting porcupine (PORCN), a protein important for Wnt secretion and biological activity. However, intraperitoneal injection of IWP-2 in restraint-stressed animals could inhibit the Wnt/β-catenin signaling activation, we observed that IWP-2 treatment abrogated 5-HT-induced disintegration of APC/Axin/GSK-3β and β-catenin degradation complex in the jejunum (Figure 4g,h) and colon (Figure 4i–k).

To investigate whether the restraint stress-induced increase in 5-HT level is dependent on Wnt transactivation, we tested whether blocking Wnt signaling inhibits the 5-HT signal. As expected, we found that in restraint-stressed mice, after inhibiting Wnt/β-catenin signaling (intraperitoneal injection of IWP-2), the number of intestinal EC cells no longer increased (Figure 1e–g), the expression level of 5-HT synthase TPH1 and 5-HT receptor HTR7 decreased significantly, and the expression level of 5-HT transporter SERT increased significantly in the colon (Figure 4l,m). Taken together, these results suggested that the enhancement of 5-HT signal induced by restraint stress was disturbed by Wnt/β-catenin signaling.

### 2.3. Intestinal Inflammation Induced by 5-HT May Be Due to the Interaction Between Wnt/β-Catenin Signal and NF-KB Signal

**Figure 5:**
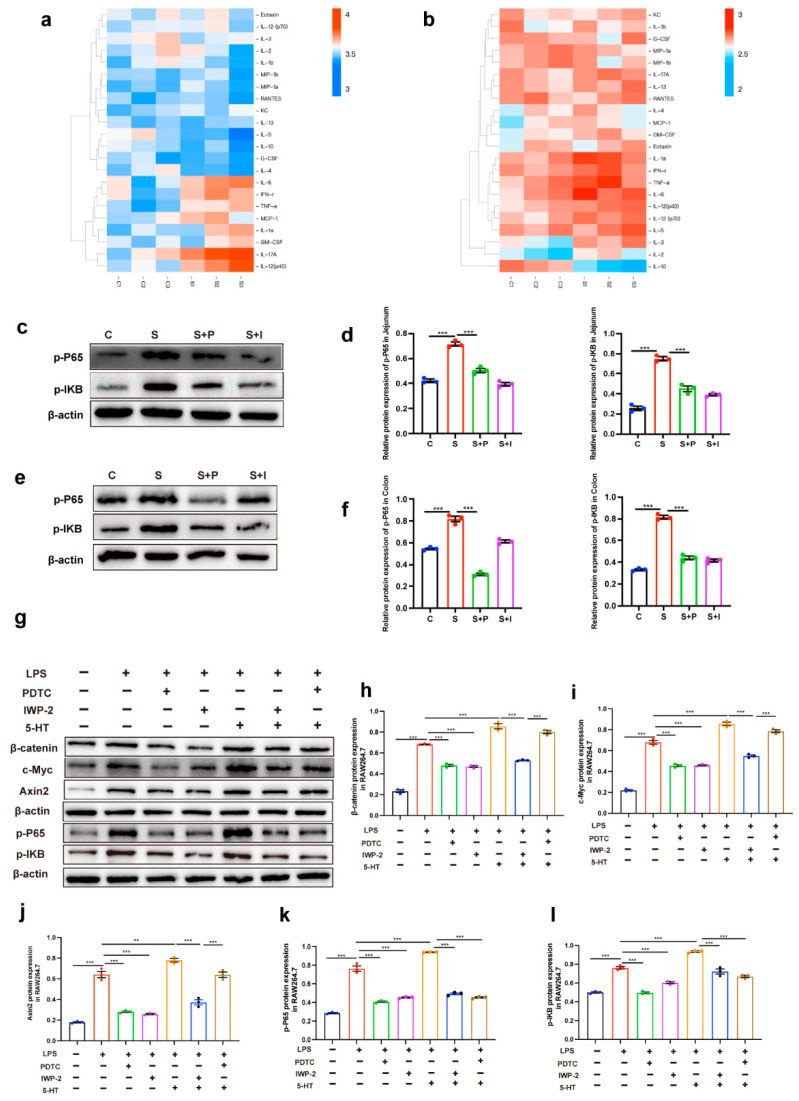
Intestinal inflammation induced by 5-HT may be due to the interaction between WNT signal and NF-KB signal. Cytokines in serum (**a**) and colon (**b**) were detected by Luminex liquid suspension chip technology (n = 3). The expression of p-P65, p-IKB and β-actin protein were examined in jejunal (**c**) and colon (**e**) tissues by Western blot, and relative protein levels were normalized to β-actin (**d**,**f**) (n = 3). RAW264.7 cells were treated with LPS, PDTC (NF-KB pathway inhibitor), IWP-2 (WNT pathway inhibitor) and 5-HT, and the expression of β-catenin, c-Myc, Axin2, p-P65, p-IKB (**g**) and β-actin protein were examined by Western blot, and relative protein levels were normalized to β-actin (**h**–**l**) (n = 3). Each sample was assayed three times. Data are presented as the mean ± SEM. Differences were assessed by ANOVA and denoted as follows: ** *p* < 0.01; *** *p* < 0.001 indicate significant difference. C: control group; S: restraint stress; S + PCPA: restraint stress + PCPA; S + IWP-2: restraint stress + IWP-2.

Inflammatory cytokines were found using Luminex liquid suspension chip technology in the serum and colon tissues of restraint-stressed mice (Table 1 and Table 2). We found that restraint stress could induce the expression of proinflammatory cytokines in serum and colon (Figure 5a,b). NF-kB is a major proinflammatory transcription factor, thereby inducing the expression of proinflammatory cytokines. Western blot analysis on the expression of p-P65 and p-IKB was significantly upregulated in jejunum and colon tissues stimulated by restraint stress (Figure 5c–f). Injecting PCPA and IWP-2 intraperitoneally into restraint-stressed mice attenuated the expression of p-P65 and p-IKB. However, treatment of the same group with pioglitazone did not result in changes to p-P65 and p-IKB expression (Figure 5c–f). We hypothesized that the activation of the NF-kB pathway was influenced by 5-HT-mediated Wnt/β-catenin signaling, so we further tested this hypothesis in vitro.

In RAW 264.7 cells treated with LPS, upregulated expression of β-catenin, c-Myc, Axin2, p-P65, and p-IKB were observed. When LPS was added following a 2 h treatment with PDTC (NF-KB inhibitors), the expression levels of β-catenin, c-Myc, Axin2, p-P65 and p-IKB significantly decreased (Figure 5g–l). This suggested that LPS may also be involved in the activation of the Wnt/β-catenin pathway. When LPS was added after IWP-2 pretreatment for 2 h, the expression levels of β-catenin, c-Myc, Axin2, p-P65 and p-IKB were significantly decreased (Figure 5g–l). This indicated that the activation of the NF-kB pathway required the presence of Wnt/β-catenin signaling. The expression of β-catenin, c-Myc, Axin2, p-P65 and p-IKB were enhanced to the greatest extent under the induction of LPS + 5-HT (Figure 5g–l). When Wnt/β-catenin signaling was blocked (IWP-2), the LPS + 5-HT-induced expression of β-catenin, c-Myc, Axin2, p-P65 and p-IKB were inhibited (Figure 5g–l). This proved once again that the presence of Wnt/β-catenin signaling was important for the activation of the NF-kB pathway. However, when pre-treatment with PDTC to suppress P65 and IKB phosphorylation blocked the LPS + 5-HT-induced effect, but the expression of β-catenin, c-Myc, and Axin2 was not significantly affected (Figure 5g–l), this indicated that the presence of 5-HT could activate Wnt/β-catenin signaling even if the NF-kB pathway was blocked.

## 3. Discussion

Exposure to psychological stress increases the risk of enteric infections [30] and bowel diseases [31]. 5-HT is a key intestinal mucosal signaling molecule that affects intestinal physiology (motor and secretory functions) to maintain gastrointestinal homeostasis [32]. EC cell hyperplasia and deregulated production of 5-HT from EC cells are associated with gastrointestinal diseases [17,18]. However, little is known about the dysregulation of EC cell homeostasis during stress-induced intestinal injury.

Our experimental findings demonstrate that restraint stress significantly compromises intestinal barrier integrity, as evidenced by increased paracellular permeability. A subset of patients with IBS showed increased intestinal permeability [33,34]. Goblet cells secrete mucin (MUC2) to defend against invading pathogenic microorganisms [35]. Stress damages the histological structure and reduces the number of goblet cells and their ability to secrete MUC2 of jejunum and colon. Furthermore, studies found that MUC2-deficient mice spontaneously develop colitis [36], and mucosal activity is significantly associated with reduced MUC2 synthesis and secretion in patients with ulcerative colitis [37], again suggesting that the mucosal barrier plays a critical role in the course of the disease. Notably, pharmacological inhibition of 5-HT biosynthesis via PCPA attenuated these stress effects, restoring goblet cell density to baseline levels and MUC2 secretion capacity. This therapeutic efficacy positions the 5-HT signaling axis as a central regulator of mucosal homeostasis during stress adaptation, potentially mediating its effects through both secretory cell maintenance and tight junction protein modulation.

Nuclear translocation of β-catenin is significantly increased in IBD [38,39], and abnormal activation of the Wnt/β-catenin pathway promotes the progression of IBD-related carcinogenesis [40,41]. We reported that stress-induced 5-HT signaling through HTR7 activated the Wnt/β-catenin pathway and promoted stem cell differentiation, leading to an increased number of EC cells, accompanied by enhanced gastrointestinal 5-HT secretion. Consistent with our findings, an increased number of EC cells and 5-HT level also occurred in early-life stress [42]. In fact, blocking 5-HT largely reduced the number of serotonin-producing EC cells in the gut of restraint-stressed mice. Studies have found that 5-HT engagement with its respective receptors participated in the regulation of Wnt/β-catenin pathway in colorectal cancer [23]. In vitro, we demonstrated that 5-HT could activate the Wnt/β-catenin pathway through HTR7 in Caco2 cells. Interaction of 5-HT with HTR7 suppressed the formation of the APC/Axin/GSK-3β-contained β-catenin degradation complex, which induces β-catenin degradation. Consistent with our prediction, intraperitoneal injection of IWP-2 into restraint-stressed mice could block the activation of the Wnt/β-catenin signaling pathway, and intestinal EC cells were no longer altered, resulting in a significantly weakened 5-HT signal. Taken together, these findings suggested that 5-HT/HTR7/Wnt/β-catenin signaling may be a valuable therapeutic target for the pathophysiological phenotype management of gastrointestinal diseases.

The Wnt signaling pathway can trigger inflammatory response, activate immune cells [43] and inflammatory signaling pathways [44,45], and promote the release of inflammatory factors [46]. In contrast, suppression of β-catenin reduced LPS-induced NF-κB activation in human bronchial epithelial cells [47]. Our experimental data showed that stress activated the NF-kB pathway, and blocking 5-HT simultaneously attenuated Wnt/β-catenin and NF-kB signals. When the Wnt/β-catenin signal was blocked, the NF-κB signal was significantly decreased.

These results demonstrate that 5-HT-mediated Wnt signaling is the primary driver of NF-κB activation under stress conditions. This regulatory axis aligns with reports of β-catenin overexpression inducing NF-κB nuclear translocation in myocardial infarction models [48], yet our work provides novel resolution of the temporal sequence. In vitro, we found that LPS treatment activated Wnt/β-catenin signaling, and when Wnt/β-catenin signaling was blocked, NF-kB signaling decreased. Consistent with our findings, endotoxemia models showed that Wnt-NF-κB crosstalk exacerbates cytokine storms and organ damage [24], but the specific mechanism has not been confirmed. In addition, we found that blocking Wnt/β-catenin signaling significantly attenuated the NF-kB response. Despite the simultaneous addition of LPS + 5-HT, no change in Nf-kB signal occurred, indicating that 5-HT induced activation of NF-kB signal was mainly mediated by Wnt/β-catenin signal. In summary, we demonstrated that the damage of intestinal barrier induced by restraint stress was mainly caused by the increase in 5-HT level, which damaged the intestinal mucosal barrier and induced the increase in intestinal inflammation through 5-HT/HTR7/Wnt/β-catenin/NF-kB pathway. However, the specific mechanism of how the interaction between WNT signaling and NF-kB signaling occurs remains unclear. These findings position the 5-HT/HTR7/Wnt/β-catenin/NF-κB axis as a promising therapeutic target for stress-exacerbated gastrointestinal disorders.

## 4. Materials and Methods

### 4.1. Animal Treatments

A total of 60 female C57BL/6J mice (age, 6–7 wk) were purchased from Vital River Laboratory Animal Technology Co. Ltd. (Beijing, China), and raised under specific pathogen-free (SPF) conventional conditions (14 h per daylighting cycle). These mice were given access to food and water ad libitum. After one week of adaptation period, 78 mice were randomly assigned to five groups: control group (C; n = 10), restraint stress group (S; n = 10), restraint stress + PCPA group (S + P; n = 10), PCPA group (P; n = 10), restraint stress + IWP-2 group (S + I; n = 10), IWP-2 group (I; n = 10). PCPA group (n = 6); IWP-2 group (n = 6); Rosiglitazone (n = 6). Mice were individually placed into ventilated transparent 50 mL plastic centrifuge tubes to limit their movements for 6 h (from 10:00 A.M. to 16:00 P.M.) for 14 consecutive days. Two hours before restraint stress, PCPA (150 mg/kg) (C6506, Sigma-Aldrich, St. Louis, MO, USA) was intraperitoneally injected into the S + P group, and IWP-2 (0.5 mg/kg) (HY-13912, MCE, NJ, SUA) was intraperitoneally injected into the S + I group. Rosiglitazone (30 mg/kg) (R2408, Sigma-Aldrich, MO, USA) was administered to the S + R group by gavage. The control group was injected with the same dose of solvent (10% DMSO, 40% PEG300, 5% Tween-80 and 45% saline). Each of the above drugs was prepared in a 10-fold stock solution with DMSO; then a 10-fold dilution was performed: add 10% DMSO (D8418, Sigma-Aldrich, MO, USA), 40% PEG300 (HY-Y0873, MCE, NJ, SUA), 5% Tween-80 (HY-Y0873, MCE, NJ, SUA), and 45% saline. In total, 100 μL was taken for intragastric and intraperitoneal injection of each mouse. PCPA and IWP-2 were administered to mice by intraperitoneal injection, while Rosiglitazone was administered to mice by gavage.

### 4.2. Ethics Approval and Consent to Participate

All animal experiments were approved by the Institutional Animal Care and Use Committee of the China Agricultural University, Beijing, China, under permit no. AW11011202-2-1 (Beijing, China). In this study, all experimental methods were performed following the China Agricultural University of Health Guide for the Care and Use of Laboratory Animals.

### 4.3. Intestinal Permeability

An in vivo permeability test was performed using the FITC-labeled dextran method to evaluate the barrier function. Food and water were withdrawn overnight, and mice were gavaged with 60 mg FITC-labeled dextran per 100 g body weight (46944; Sigma-Aldrich Co., Ltd., Shanghai, China). Serum was collected 5 h after FD-4 gavage, and the fluorescence intensity of each sample was measured (excitation, 492 nm; emission, 525 nm). FITC-dextran was diluted in PBS to form a standard curve. Calculation of FITC-dextran concentration in serum using a standard curve.

### 4.4. RNA Isolation and RT–qPCR Analysis

The intestinal tissues were isolated from the experimental mice and homogenized in TRIzol Reagent, and total RNA was isolated according to the manufacturer’s instructions. RNA (1 μg) was reverse transcribed into complementary DNA using HiScript II Select qRT SuperMix (R312-02, Vazyme, Naijing, China). Real-time quantitative PCR (qPCR) was performed using gene-specific primer sets and SYBR green master mix (Q141-0, Vazyme). Changes in fluorescence were monitored on a OneStep Plus instrument (Applied Biosystems, Foster City, CA, USA). The primers used are shown in Table 3.

### 4.5. Cell Treatment

Caco2 (HTB-37, ATCC) and RAW 264.7 (TIB-71, ATCC) cells were cultured in Dulbecco’s modified Eagle’s medium (DMEM) (Vivacell, Shanghai, China) supplemented with 10% fetal bovine serum (FBS) and penicillin/streptomycin (100 ng/mL) at 37 °C in a humidified atmosphere with 5% carbon dioxide. The cells were cultured in 48-well culture plates (1 × 10^4^ cells/mL) and 6-well culture plates (1 × 10^6^ cells/mL). Cells were allowed to attach overnight, which were then washed twice with PBS and subsequently replenished with the serum-free media. In the experiments with 5-HT (H9523CAS, Sigma-Aldrich, MO, USA) and SB-269970 (HY-15370, MCE, NJ, SUA), cells were incubated with 20 μm SB269970 (HTR_7_ antagonist) for 2 h and then incubated with 10 μm 5-HT for 24 h. Cells were stimulated with 20 μm IWP-2 (Wnt inhibitors) (HY-13912, MCE, NJ, SUA), 5 μM PDTC (an NF-κB antagonist) (HY-18738, MCE, NJ, SUA), LPS (10 nM) (L8880, Solarbio, Beijing, China) or the medium alone for 24 h. The culture medium was collected, and the total cellular protein was extracted for biochemical and Western blot analysis.

### 4.6. Cellular Immunofluorescence Analysis

Immunofluorescence was used for detecting β-catenin in Caco2 cells. Cells were fixed in 4% paraformaldehyde, washed with PBS (three washes of 5 min each), permeabilized in 0.1% Triton X-100 (9002-93-1, Sigma-Aldrich, MO, USA) for 10 min, and washed with PBS (three washes of 5 min each). Cells were incubated for 1.5 h with normal goat serum (5%, diluted in PBS) at 37 °C to saturate non-specific binding sites. Incubation was carried out with the primary antibody, then incubation with the corresponding secondary antibody at room temperature for 60 min. The nucleus was stained by DAPI (C0065, Solarbio, Beijing, China) and washed three times with PBS. 

### 4.7. Histological Analysis

The intestine was washed with PBS, fixed with 4% paraformaldehyde, and embedded in paraffin. The tissue was then sectioned (5 μm), and the sections were dewaxed to water. Intestinal tissue sections were stained with hematoxylin and eosin (H&E). Tissues were stained with Alcian Blue Periodic acid Schiff/AB-PAS. Goblet cells were enumerated in a 100 μm stretch, at least 30 random fields in six sections of each sample from AB-PAS staining were photographed at 400× magnification with a microscope (BX51; Olympus, Japan). Immunohistochemistry to stain for chromogranin A (sc-393941, Santa, Cruz, Dallas, TX, USA) and MUC2 (ab272692, Abcam, Cambridge, CA, USA) in paraffin intestinal sections. Intestinal tissue sections were incubated with the rabbit anti-chromogranin A and the rabbit anti-MUC2 overnight at 4 °C. After, the sections were washed with PBS (pH 7.4) and incubated sequentially with biotinylated goat anti-rabbit IgG secondary antibodies for 2 h. Next, following the washing, they were incubated sequentially with 1:300 HRP–streptavidin for 2 h. After treatment with diaminobenzidine (DAB) Kit (C0065, Solarbio, Beijing, China), it showed an immune response, and hematoxylin counterstained the nucleus for 5 min. The average integrated optical density (IOD) of the positive cells was measured by ImageJ (National Institutes of Health, Bethesda, MD, USA). HTR7, LGR5, and β-catenin in paraffin intestinal sections. The tissues were incubated overnight at 4 °C with the following primary antibodies: rabbit anti-HTR7 antibody (DF13323, Affinity, OH, USA), rabbit anti-LGR5 (DF2816, Affinity, OH, USA), and rabbit anti-β-catenin (66379-1-Ig, Proteintech, Wuhan, China). Subsequently, the tissues were washed on a decolorization shaker with PBS and then incubated with the corresponding secondary antibody, Goat Anti-Rabbit Alexa fluor 594 (ab150080, Abcam, Cambridge, CA, USA) and Goat Anti-Mouse Alexa fluor 488 (ab150113, Abcam, Cambridge, CA, USA), at room temperature for 60 min. The sections were photographed with a Nikon Eclipse TE 2000S inverted microscope (Nikon Instruments, Inc, Melville, NY, USA). The numbers of positively stained puncta were counted using ImageJ software (National Institutes of Health, Bethesda, MD, USA).

### 4.8. Western Blotting

Total proteins were extracted from intestinal tissues and cell lines using RIPA lysis buffer (R0010, Solarbio Life Sciences, Beijing, China) with protease and phosphatase inhibitors for 30 min. Protein concentration were determined by BCA protein quantification kit, and the proteins were separated by 10–15% SDS-PAGE, and then electro transferred to a polyvinylidene fluoride membrane. The membrane was then blocked with 5% skimmed milk in Tris-buffered saline with Tween 20 (TBST) at room temperature for 1.5 h. Subsequently, the membrane was incubated overnight at 4 °C with the antibodies: rabbit anti-Claudin-3 antibody (1:1000, ab15102, Abcam, Cambridge, CA, USA), rabbit anti-Claudin-1 antibody (1:3000, ab15098, Abcam, Cambridge, CA, USA), rabbit anti-Occludin antibody (1:1000, ab216327, Abcam, Cambridge, CA, USA), rabbit anti-TPH1 antibody (1:1000, ab52954, Abcam, Cambridge, CA, USA), rabbit anti-SERT antibody (1:1000, 19559-1-AP, Proteintech, Wuhan, China), rabbit anti-HTR7 antibody (1:1000, DF13323, Affinity, OH, USA), mouse anti-β-catenin (1:8000, 66379-1-AP, Proteintech, Wuhan, China), rabbit anti-APC antibody (1:1000, ab40778, Abcam, Cambridge, CA, USA), rabbit anti-Axin1 antibody (1:1000, C76H11, Cell Signaling Technology, Beverly, MA, USA), rabbit anti-GSK3-β antibody (1:1000, SC-377213, Santa Cruz, Dallas, TX, USA), rabbit anti-TCF3 antibody (1:2000, 21242-1-AP, Proteintech, Wuhan, China), rabbit anti-Phospho-IkB (1:1000, ab133462, Abcam, Cambridge, CA, USA), rabbit anti-Phospho-NF-kB p65 (1:1000, ab76302, Abcam, Cambridge, CA, USA), mouse anti-β-actin antibody (1:10,000, 66009-1-lg, Proteintech, Wuhan, China), rabbit anti-Lgr5 (1:1000, DF2816, Affinity Biosciences, Jiangsu, China), rabbit anti-Axin2 (1:1000, 20540-1-AP, Proteintech, Wuhan, China), and mouse anti-c-Myc (1:1000, sc-40, Santa Cruz, Dallas, TX, USA) overnight at 4 °C. Then, they were incubated with horseradish peroxidase-conjugated goat anti-mouse IgG (1:10,000, SA00001-1, Proteintech, Wuhan, China) (for β-actin) or goat anti-rabbit IgG (1:10,000, SA00001-2, Proteintech, Wuhan, China) for 2 h at room temperature. Immunoblots were performed using an ECL Western blot kit (CW0049; CoWin Biotech Co., Inc, Beijing, China). The bands on the blots were scanned and analyzed by ImageJ software (National Institutes of Health, Bethesda, MD, USA).

### 4.9. Measurement of Serotonin Concentration in Intestinal Tissues

Serotonin concentrations were measured using competitive enzyme-linked immunosorbent assay kits (J24411, Gilead Biotechnology Co., Ltd.). The measurement range of serotonin concentration was expressed as 0–80 nmol/L. All assays were performed according to the kit manufacturer’s instructions.

### 4.10. Coimmunoprecipitation Assay for Protein–Protein Binding Interactions

Coimmunoprecipitation experiments were performed using an immunoprecipitation kit (abs955, Absin, Shanghai, China) according to the manufacturer’s instructions. The cells were harvested, and after removing the medium, the cells were washed three times with precooled PBS. Cells were lysed in a homogenizer with the lysis buffer provided in the kit, lysed at 4 °C for 30 min, and then centrifuged at 10,000× *g* for 10 min at 4 °C.

The precipitate was removed from the cell lysate after centrifugation at 14,000× *g* for 10 min, and the supernatant was precleared with protein A/G beads. Immunoprecipitation of the complexes was performed by incubating the cellular lysates with β-catenin antibody at 4 °C overnight. After adding protein A/G bead slurry and incubating for 1 h at 4 °C, the beads were collected by centrifugation at 12,000× *g* for 1 min and washed three times with the wash buffer provided with the kit. Finally, samples were subjected onto SDS–PAGE gels and detected by immunoblotting.

### 4.11. Statistical Methods

Data were presented either as mean ± standard errors. Data analysis was performed by GraphPad Prism 6.0 program (GraphPad Software Inc., La Jolla, CA, USA). The significant differences between the control and stress mice in serotonin content between different intestinal segments were evaluated by an independent sample *t* test (two-tailed test). One-way ANOVA and Tukey’s multiple comparison test were used to compare multiple groups. *p* < 0.05 was considered statistically significant.

## Figures and Tables

**Table 1:** Expression of inflammatory factors in serum.

Cytokines	C	S	*p* Value
IL-1α	11.92 ± 0.5486	14.19 ± 1.237	0.1694
IL-1β	12.57 ± 0.8827	11.72 ± 0.6573	0.4830
IL-2	13.15 ± 0.8552	12.38 ± 0.9782	0.5866
IL-3	13.80 ± 0.5379	12.62 ± 0.3701	0.1469
IL-6	12.45 ± 0.9461	16.11 ± 0.7175	0.0369 *
IL-12(p40)	12.42 ± 0.4264	16.85 ± 1.049	0.0173 *
IL-12 (p70)	12.60 ± 0.3567	12.74 ± 0.6593	0.8590
IL-17A	13.29 ± 0.9735	18.25 ± 0.6980	0.0144 *
Eotaxin	12.92 ± 0.5740	12.73 ± 0.6233	0.8304
GM-CSF	12.36 ± 0.5398	13.34 ± 1.024	0.4443
IFN-γ	12.14 ± 0.9250	15.65 ± 0.6022	0.0336 *
KC	11.68 ± 0.4232	12.55 ± 0.5199	0.2660
MCP-1	12.55 ± 0.5398	14.53 ± 0.8864	0.1288
MIP-1a	11.20 ± 0.2905	11.76 ± 0.6532	0.4797
MIP-1b	11.64 ± 0.3495	11.68 ± 0.6964	0.9618
RANTES	11.44 ± 0.4923	11.31 ± 0.5398	0.8694
TNF-α	11.25 ± 0.4965	14.39 ± 0.4431	0.0092 **
IL-13	11.72 ± 0.4328	11.56 ± 0.3449	0.7822
G-CSF	11.67 ± 0.6958	10.86 ± 0.2745	0.3347
IL-4	12.63 ± 0.7342	10.72 ± 0.2379	0.0686
IL-5	13.15 ± 0.6566	10.40 ± 0.5798	0.0350 *
IL-10	12.46 ± 0.3397	10.88 ± 0.5998	0.0834

* *p* < 0.05, ** *p* < 0.01.

**Table 2:** Expression of inflammatory factors in colonic tissue.

Cytokines	C	S	*p* Value
IL-1α	10.50 ± 0.5220	13.26 ± 0.6310	0.0281 *
IL-1β	9.416 ± 1.193	9.748 ± 0.6097	0.8166
IL-2	6.884 ± 0.5568	8.704 ± 0.5932	0.0889
IL-3	7.918 ± 0.5836	10.80 ± 0.5604	0.0235 *
IL-6	10.22 ± 0.6225	12.91 ± 0.7110	0.0466 *
IL-12(p40)	9.820 ± 0.5456	11.95 ± 0.1158	0.0188 *
IL-12 (p70)	9.715 ± 0.5167	11.56 ± 0.3348	0.0399 *
IL-17A	9.931 ± 0.6067	11.21 ± 0.3143	0.1340
Eotaxin	9.387 ± 0.7543	9.657 ± 0.8402	0.8228
GM-CSF	8.559 ± 0.3482	9.962 ± 0.5812	0.1072
IFN-γ	10.16 ± 0.3184	12.02 ± 0.4696	0.0303 *
KC	9.754 ± 0.6356	9.787 ± 0.5508	0.9709
MCP-1	8.506 ± 0.8345	8.927 ± 0.5487	0.6950
MIP-1a	10.82 ± 0.5459	9.910 ± 0.5770	0.3155
MIP-1b	10.80 ± 0.5945	9.560 ± 0.8817	0.3072
RANTES	10.82 ± 0.6647	10.53 ± 0.7689	0.7914
TNF-α	10.40 ± 0.5129	12.89 ± 0.5803	0.0321 *
IL-13	9.940 ± 0.5399	10.92 ± 0.6066	0.2927
G-CSF	10.59 ± 0.3494	9.887 ± 1.094	0.5732
IL-4	8.710 ± 0.6097	9.252 ± 0.8162	0.6231
IL-5	10.77 ± 0.5018	11.45 ± 0.8444	0.5284
IL-10	10.85 ± 0.5929	5.920 ± 0.5837	0.0041 **

* *p* < 0.05, ** *p* < 0.01.

**Table 3:** Primers for real-time PCR.

Gene Name	Forword Sequences (5′-3′)	Reverse Sequences (5′-3′)
***β-catenin***	TGACACCTCCCAAGTCCTTT	TTGCATACTGCCCGTCAAT
***Axin2***	GAGAGTGAGCGGCAGAGC	CGGCTGACTCGTTCTCCT
***Cd44***	TCGATTTGAATGTAACCTGCCG	TCGATTTGAATGGATGTAACCTGCA
***Myc***	ATGCCCCTCAACGTGAACTTC	CGCAACATAGGATGGAGAGCA
***Lgr5***	CCTACTCGAAGACTTACCCAGT	GCATTGGGGTGAATGATAGCA
***Sox9***	CTGGAGGCTGCTGAACGAGAG	CGGCGGACCCTGAGATTGC
***Olfm4***	CAGCCACTTTCCAATTTCACTG	GCTGGACATACTCCTTCACCTTA
***Htr7***	TGCGGGGAGCAGATCAACTA	GACAAAGCACACCGAGATCAC
***Htr4***	GGCTATATCAATTCGGGGTTGAA	GTGTATGGGCAATTTCTCCAGTT
***Htr3***	CCTGGCTAACTACAAGAAGGGG	TGCAGAAACTCATCAGTCCAGTA
***Htr2***	TAATGCAATTAGGTGACGACTCG	GCAGGAGAGGTTGGTTCTGTTT
***Tph1***	AACTTTCACACTTCAGATTCACC	ATAGGCCGTCTCTGAGGAAC
***Tph2***	GGTTGTCCTTGGATTCTGCTG	GCCTGGATTCGATATGAAGCAT
***Slc6a4***	TATCCAATGGGTACTCCGCAG	CCGTTCCCCTTGGTGAATCT
***Muc2***	AGGGCTCGGAACTCCAGAAA	CCAGGGAATCGGTAGACATCG
***Tff3***	TTGCTGGGTCCTCTGGGATAG	TACACTGCTCCGATGTGACAG
***Klf3***	GAAGCCCAACAAATATGGGGT	GGACGGGAACTTCAGAGAGG
***Muc2***	AGGGCTCGGAACTCCAGAAA	CCAGGGAATCGGTAGACATCG
***Tff3***	TTGCTGGGTCCTCTGGGATAG	TACACTGCTCCGATGTGACAG
***Klf3***	GAAGCCCAACAAATATGGGGT	GGACGGGAACTTCAGAGAGG
***Tff3***	TTGCTGGGTCCTCTGGGATAG	TACACTGCTCCGATGTGACAG
***Klf3***	GAAGCCCAACAAATATGGGGT	GGACGGGAACTTCAGAGAGG
***Gapdh***	AGCTTGTCATCAACGGGAAG	TTTGATGTTAGTGGGGTCTCG

## Data Availability

All data generated or analyzed during this study are included in this published article.

## Supplementary Materials

The following supporting information can be downloaded at: https://www.mdpi.com/article/10.3390/ijms26094021/s1. References [49,50,51,52] are cited in the Supplementary Materials.
