# Supplementary material for: Restraint Stress Disrupted Intestinal Homeostasis via 5-HT/HTR7/Wnt/β-Catenin/NF-kB Signaling
Source: Int J Mol Sci. 2025 Apr 24;26(9):4021. doi: 10.3390/ijms26094021 (PMC12071331; doi:10.3390/ijms26094021)
Supplement: Supplementary file 1 [file ijms-26-04021-s001.zip › ijms-3435079-supplementary.pdf]

Supplementary Material

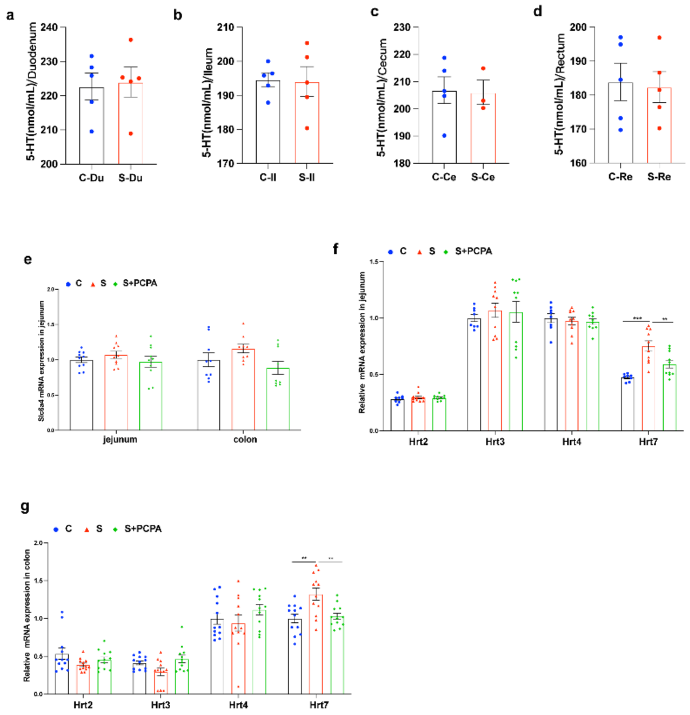

Figure S1. Changes in intestinal 5-HT related indicators; (a–d) Changes of 5-HT levels in intestine of mice (n=5). (e) Changes of Slc6a4 mRNA levels in jejunum and colon tissues of mice (n=10). (f-g) The expression of Htr2, Htr3, Htr4 and Htr7 were measured by real-time qPCR in jejunum and colon tissues of mice (n = 10). Each sample was assayed three times. Data are presented as the mean ± SEM. Differences were assessed by ANOVA and denoted as follows: **P < 0.01; ***P < 0.001 indicate significant difference. C: control group; S: restraint stress; S+PCPA: restraint stress + PCPA.

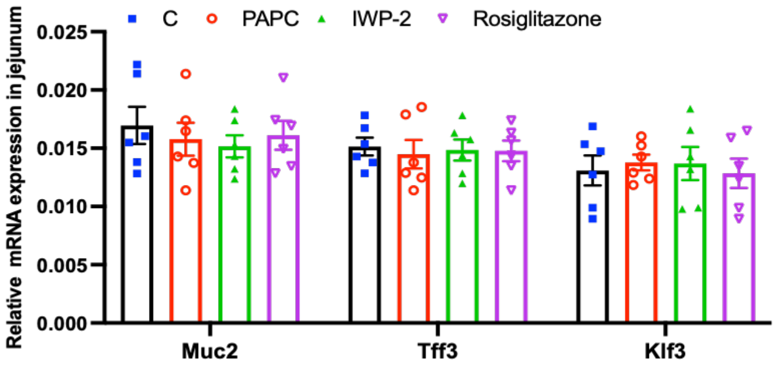

Figure S2. The mRNA levels of Muc2 (the main component of mucous layer), Tff3 (promoting mucosal repair and protection), and Klf3 (participating in the transcription of barrier function) in jejunum were detected by qPCR (n=6). Data are presented as the mean ± SEM. C: control group;

PAPC：Intraperitoneal injection of PCPA group; IWP-2: Intraperitoneal injection of IWP-2 group; Rosiglitazone: Rosiglitazone group by gavage.

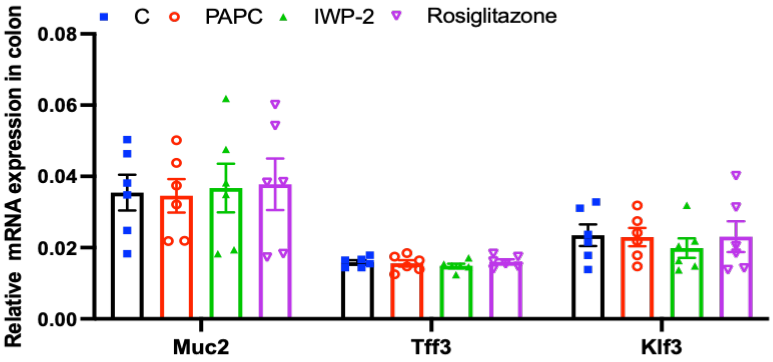

Figure S3. The mRNA levels of Muc2 (the main component of mucous layer), Tff3 (promoting mucosal repair and protection), and Klf3 (participating in the transcription of barrier function) in Colon were detected by qPCR (n=6). Data are presented as the mean ± SEM. C: control group; PAPC：Intraperitoneal injection of PCPA group; IWP-2: Intraperitoneal injection of IWP-2 group; Rosiglitazone: Rosiglitazone group by gavage.

The explanatory notes regarding the construction of the restraint stress model:

Transparent 50 mL plastic centrifuge tubes are used and undergo ventilation treatment. Adult mice (such as the C57BL/6 strain, with a body length of 8-10 cm) are unable to turn around or move freely in a 50 mL tube with a diameter of approximately 3 cm and a length of about 11.5 cm. However, they can still maintain their basic respiratory needs. The design of ventilation holes avoids the risk of asphyxiation, which is in line with the standards of animal welfare. The transparent tube may increase psychological stress (such as the perception of environmental threats), while physical restraint directly induces physiological stress. The combination of these two factors makes the situation more similar to a real stress scenario [1].

Duration of restraint: The mice are restricted from moving for 6 hours per day, and this treatment is carried out continuously for 14 days. A duration shorter than 6 hours may not be sufficient to activate the chronic stress pathway (such as the hypothalamic-pituitary-adrenal axis, HPA axis), while a duration exceeding 6 hours may lead to excessive stress (such as immunosuppression or tissue damage). 6 hours is a commonly used threshold for the chronic stress model. Mice generally need 3-5 days to adapt to short-term stress, and a continuous treatment for 14 days allows for the observation of chronic effects (such as an increase in corticosterone levels and depression-like behaviors) [2].

Time period of restraint: The restraint is implemented from 10:00 am to 4:00 pm during the light phase (the inactive period of mice). Mice are nocturnal animals, and their active period is usually during the dark phase. Restraining them during the light phase (inactive period) can reduce physical injuries caused by struggling. At the same time, it simulates "passive stress" (such as sleep disruption or rest deprivation), enhancing psychological stress. Restraining the mice at a fixed time period (the same time every day) reduces the variable of circadian rhythm interference and improves the consistency of the experimental results [3].

Necessity of continuous treatment for 14 days: Short-term stress (less than 7 days) mainly elicits an acute response (such as a transient increase in corticosterone levels), while a 14-day treatment can lead to persistent HPA axis hyperactivity, hippocampal neuronal atrophy, and other chronic pathological changes. Depression-like behaviors (such as an increase in the immobility time during the forced swim test) or cognitive deficits require at least 2 weeks of induction [4].

1. Yang D, Sun Y, Wen P, Chen Y, Cao J, Sun X, Dong Y: Chronic Stress-induced Serotonin Impairs Intestinal Epithelial Cell Mitochondrial Biogenesis via the AMPK-PGC-1α Axis. Int J Biol Sci 2024, 20:4476-4495.

2. Wei W, Liu Y, Hou Y, Cao S, Chen Z, Zhang Y, Cai X, Yan Q, Li Z, Yuan Y, et al: Psychological stress-induced microbial metabolite indole-3-acetate disrupts intestinal cell lineage commitment. Cell Metab 2024, 36:466-483.e467.

3. Fonken LK, Nelson RJ: The effects of light at night on circadian clocks and metabolism. Endocr Rev 2014, 35:648-670.

4. Chiba S, Numakawa T, Ninomiya M, Richards MC, Wakabayashi C, Kunugi H: Chronic restraint stress causes anxiety- and depression-like behaviors, downregulates glucocorticoid receptor expression, and attenuates glutamate release induced by brain-derived neurotrophic factor in the prefrontal cortex. Prog Neuropsychopharmacol Biol Psychiatry 2012, 39:112-119.
